# SKPDB: a structural database of shikimate pathway enzymes

**DOI:** 10.1186/1471-2105-11-12

**Published:** 2010-01-07

**Authors:** Helen A Arcuri, Geraldo FD Zafalon, Evandro A Marucci, Carlos E Bonalumi, Nelson JF da Silveira, José M Machado, Walter F de Azevedo, Mário S Palma

**Affiliations:** 1 CEIS/Departamento de Biologia, Instituto de Biociências, UNESP, Rio Claro, São Paulo, Brasil; 2 Departamento de Ciência da Computação e Estatística, UNESP/IBILCE, São José do Rio Preto, São Paulo, Brasil; 3 Departamento de Ciências Exatas, UNIFAL, Alfenas, Minas Gerais, Brasil; 4 Faculdade de Biociências, PUCRS, Porto Alegre, Rio Grande do Sul, Brasil

## Abstract

**Background:**

The functional and **s**tructural characterisation of enzymes that belong to microbial metabolic pathways is very important for structure-based drug design. The main interest in studying shikimate pathway enzymes involves the fact that they are essential for bacteria but do not occur in humans, making them selective targets for design of drugs that do not directly impact humans.

**Description:**

The ShiKimate Pathway DataBase (SKPDB) is a relational database applied to the study of shikimate pathway enzymes in microorganisms and plants. The current database is updated regularly with the addition of new data; there are currently 8902 enzymes of the shikimate pathway from different sources. The database contains extensive information on each enzyme, including detailed descriptions about sequence, references, and structural and functional studies. All files (primary sequence, atomic coordinates and quality scores) are available for downloading. The modeled structures can be viewed using the Jmol program.

**Conclusions:**

The SKPDB provides a large number of structural models to be used in docking simulations, virtual screening initiatives and drug design. It is freely accessible at http://lsbzix.rc.unesp.br/skpdb/.

## Background

**Figure 1:**
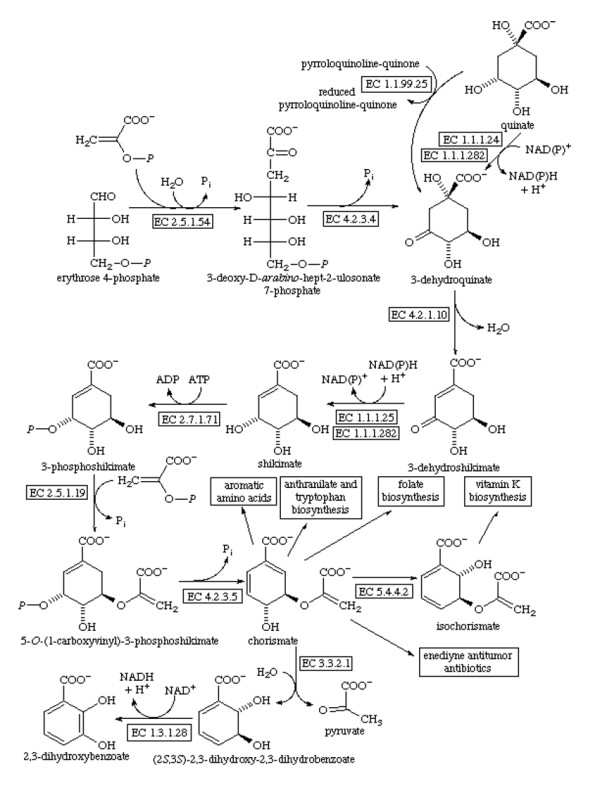
**The sequence of seven metabolic steps in the shikimate pathway, from phosphoenolpyruvate and erythrose 4-phosphate to chorismate, adapted from the site: http://www.chem.qmul.ac.uk/iubmb/enzyme/reaction/misc/shikim.html**.

The functional and structural characterisation of enzymes belonging to microbial metabolic pathways is very important for structure-based drug design [1]. The main interest in the study of shikimate pathway enzymes (Figure 1) involves the fact that they are not present in humans, but are essential for algae, higher plants, bacteria, fungi, and apicomplexan parasites. This makes them attractive targets for development of new antimicrobial drugs and decreases the impact of drugs in humans [2,3].

This pathway links the metabolism of carbohydrates to the biosynthesis of aromatic compounds through seven metabolic steps, where phosphoenolpyruvate (PEP) and erythrose 4-phosphate are converted to chorismate, which in turn is the common precursor for synthesising a series of aromatic compounds, naphtoquinones, menaquinones, and mycobactins [3,4].

Inhibition of the shikimate pathway has been effective in controlling bacterial growth [5], and in mycobacteria, this pathway has been shown to be essential for the viability of *Mycobacterium tuberculosis *[6-8].

The functional and structural characterisation of a protein sequence is one of the most frequent problems in structural molecular biology. This task is usually facilitated by an accurate three-dimensional (3D) structure of the studied protein, which is best determined by experimental methods such as X-ray crystallography and NMR spectroscopy [9]. In the absence of an experimentally determined 3D structure, the modeling (comparative or by homology) can sometimes provide a useful 3D model for a target protein [10]. In the present work, we used comparative modeling at a large scale for predicting protein structures through the program MODELLER [11].

The automation of large-scale comparative modeling involves assembling a software pipeline, which consists of modules for fold assignment, template selection, target-template alignment, model generation, and model evaluation. Computer programs for these individual operations already exist, and it may seem trivial to combine them [11,12]. One example of large-scale comparative modeling for complete genomes has been described for sequences encoded in the *Mycobacterium tuberculosis *and *Xylella fastidiosa *genomes in the DBMODELING database [13,14]. The challenge in large-scale comparative modeling is to build an automated, fast, robust, sensitive, and accurate pipeline applicable to whole genomes; such a pipeline should perform at least as well as a human expert on individual proteins.

However, since the accuracy of structural models is highly dependent on sequence identity between template and target, it is necessary to make clear to the user that only models presenting high structural quality should be used in such efforts. Molecular modeling of these enzymes generated the SKPDB database, in which all structural models were built by using alignments presenting more than 30% sequence identity, generating models with medium and high accuracy [10,15].

SKPDB is a relational database of protein structure predicted by comparative modeling or solved by X-ray crystallography, applied to the study of shikimate pathway enzymes of microorganisms and plants. This database is freely accessible for all users on the Web, providing us with a large number of structural models for use in structure-based virtual screening and molecular docking analysis. Furthermore, SKPDB also provides a docking interface, which allows the user to carry out geometric docking simulations against the molecular models available in the database.

## Construction and Content

### Molecular modeling in large scale

Homology modeling is usually the method of choice when there is a clear relationship of homology between the sequences of a target protein and at least one experimentally determined three-dimensional structure. This computational technique is based on the assumption that tertiary structures of two proteins will be similar if their sequences are related, and it is the approach most likely to give accurate results [16].

The number of protein sequences that can be modeled and the accuracy of the predictions are increasing steadily due to the growth in the number of experimentally determined protein structures and because of the improvements in the modeling software. It is currently possible to model with useful accuracy significant parts of approximately one half of all known protein sequences [17].

The molecular modeling in this work was performed by the MODELLER version 9v4 [10,18] program, which is a computer program for comparative protein structure modeling http://salilab.org/modeller. The program extracts atom-atom distance and dihedral angle restraints on the target from the template structure, and combines them with general rules of protein structure such as bond length and angle preferences. The model is then calculated by an optimisation procedure that minimises violations of the spatial restraints [11]. In the simplest case, the input is an alignment of a sequence to be modeled with the template structures, the atomic coordinates of the templates and a short script file. MODELLER then automatically calculates a model containing all non-hydrogen atoms, without any user intervention and within minutes on a processor [19].

The MODELLER program was completely automated to calculate comparative models for a large number of protein sequences, by using many different template structures and sequence-structure alignments [12,16,17]. Sequence-structure matches are established by aligning SALIGN [20] sequence profile of the target sequence against each of the template sequences extracted from PDB [21]. Significant alignments covering distinct regions of the target sequence are chosen for modeling. Models are calculated for each one of the sequence-structure matches by using MODELLER [11]. The models consist of coordinates for all non-hydrogen atoms in the modeled part of a protein [16]. For each enzyme in the SKPDB, a total of 1000 models were generated and the final models were selected based on stereochemical quality and objective function by MODELLER. The final models were then evaluated by composite model quality criteria (see topic Analysis tools).

**Figure 2:**
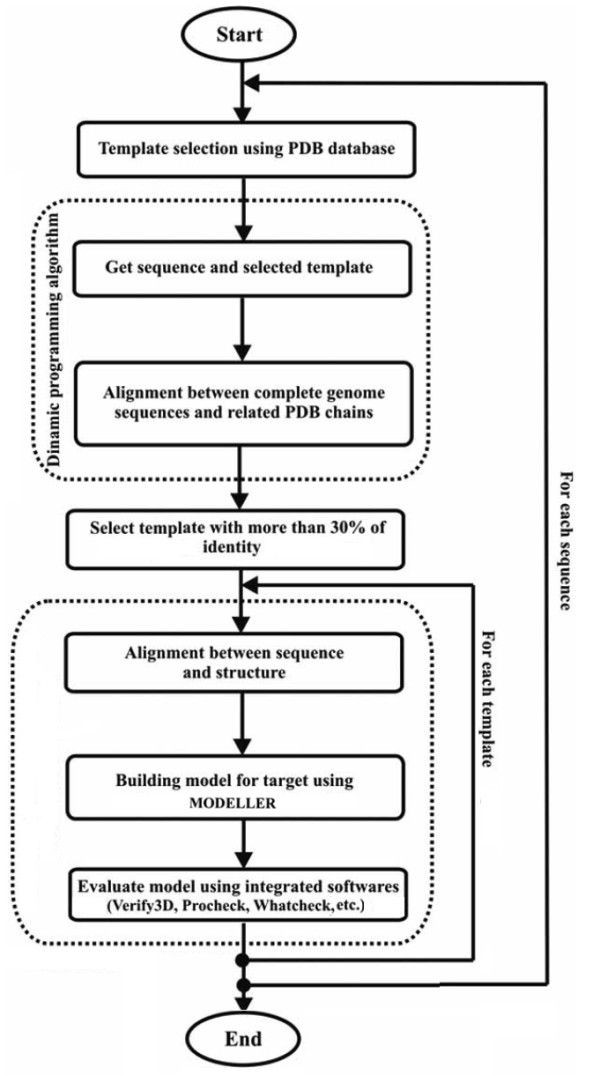
**The flowchart describing all steps of comparative modeling in large scale, used in the model predictions for SKPDB, adapted from **[27].

The MODELLER program was parallelised on a Beowulf cluster with 16 nodes (AMD Athlon 2100+ BioComp. São José do Rio Preto, SP, Brazil). It controls the distribution of the MODELLER jobs on the Beowulf cluster using a C language library, message passing interface (MPI). It allows parallel MODELLER execution and decreases the processing time of the modeling process. Figure 2 shows a schematic drawing of automated comparative modeling in large scale used in the modeling process for SKPDB.

### Analysis tools

Difficult cases in homology modeling correspond to protein sequences that only possess distant homologues of known structure. In such cases, incorrect alignment can lead to regions of a model that have significant structural errors. The quality of the predicted model determines the information that can be extracted from it. Thus, estimating the accuracy of 3D protein models is essential for interpreting them. The model can be evaluated as a whole as well as in the individual regions [13].

The overall stereochemical quality and the evaluation of the final model were performed by the programs PROCHECK [22] and WHATCHECK. These programs were used to check bond lengths, bond angles, peptide bonds and side-chain ring planarities, chirality, main-chain and side-chain torsion angles. Another quality score used in the analysis of the structural model was the G-factor, which is essentially just a log-odds score based on the observed distributions of the stereochemical parameters performed by the program PROCHECK [22]. The root mean square deviations (RMSD) from ideal geometries for bond lengths, bond angles, dihedrals and impropers were extracted for each model by using the program X-PLOR [23], and the program VERIFY-3D was used to measure the compatibility of a protein model with its sequence by using a 3D profile [24,25]. These programs were used to assess the quality of the available models and can be accessed by any user in the SKPDB web page for each enzyme.

### Web SKPDB platform

The SKPDB is implemented on Apache server 2 http://apache.org/ with Fedora 9 http://fedoraproject.org/ as an operating system. The MySQL server version 4.0.20 http://www.mysql.com is used in SKPDB to store, retrieve and manage the data. All scripts for data querying and retrieving were written in PERL/CGI version 5.8.4 http://www.perl.com/, and JAVA http://java.sun.com. The web interfaces are designed using HTML language with some scripts in JavaScript, and they rely on Cascading Style Sheet (CSS) support. The modeled structures can be viewed with Jmol http://jmol.sourceforge.net/, which is an open source software suite. Results are displayed in html format.

### Data Source

**Figure 3:**
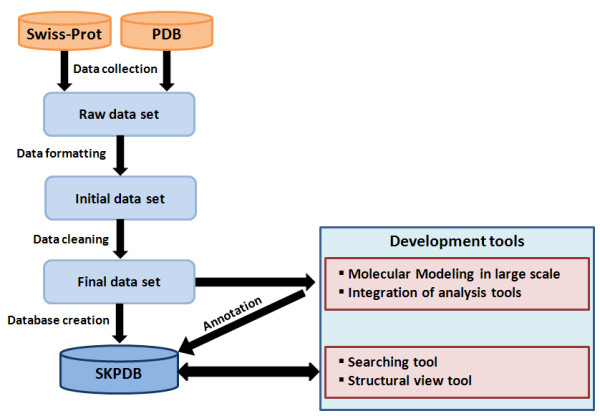
**The construction of the SKPDB database**.

All entries in SKPDB were sourced from Swiss-Prot/UniprotKB [26] protein sequence database and PDB [21] protein structure database. Initially, exhaustive queries were made to Swiss-Prot/UniprotKB, returning more than 10.000 enzymes of shikimate pathways from different organisms. The process of building SKPDB is shown in figure 3. The enzyme data were then filtered to exclude redundancy, errors, and incomplete data. Then the data were included into a single composite non-redundant database.

Currently, SKPDB has 8902 enzymes of the shikimate pathway from microorganisms and plants. The majority of the protein structures (5477 entries) were predicted by comparative modeling, though the database also includes 53 protein structures solved by crystallography and 3372 proteins without any structure solved/modeled.

### SKPDB description

SKPDB is a relational database of protein structures predicted by comparative modeling or solved by crystallography, applied to the study of shikimate pathway enzymes. Each entry in SKPDB provides information about a given enzyme, including: (1) a detailed description of the enzyme, (2) the primary sequence of the enzyme, (3) the structure model of the enzyme, (4) the chemical properties of the enzyme, (5) references about the enzyme, and (6) comments and miscellaneous information. All files (primary sequence, atomic coordinates and quality values) are available for downloading. This database is available for all users on the Web, providing a large amount of structural models to be used in virtual screening initiatives and molecular docking.

The SKPDB is regularly updated with the addition of new data and tools about shikimate pathway enzymes. A click on the links opens a new window that displays more detailed information for the selected enzyme, in different biological databases such as Swiss-Prot/UniprotKB, PDB, KEGG, BRENDA, IUMB, and PUBMED, among others. The enzyme records page contains primary sequence and structure of the model, information about alignment, analysis of target models such as PROCHECK, G-factor and the values of the RMSD from ideal geometry.

### Description table content in SKPDB

**Figure 4:**
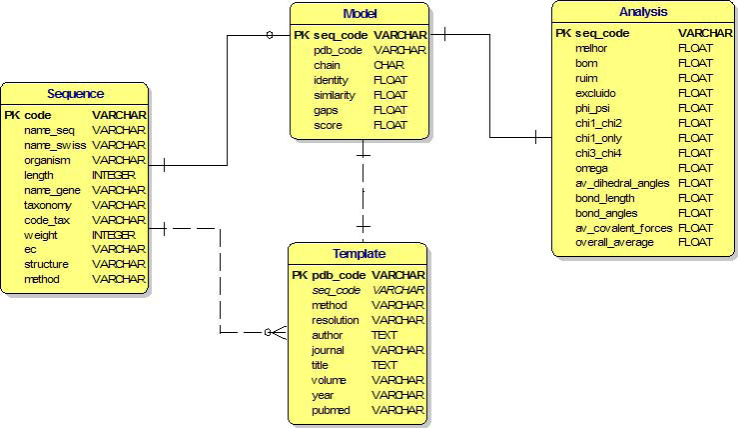
**This diagram describes the entity relationships for database tables**.

For data storing, a database was built that contained the following tables: sequence, model, template and analysis. The data were included in the tables through the scripts in Perl and the use of language specific modules (DBD:: mysql) that allow for interaction with the database, as shown in figure 4. The database is queried from an html client using Perl-CGI programming, which displays the records as dynamically generated web pages in different frames.

## Utility and Discussion

### How to use the SKPDB?

**Figure 5:**
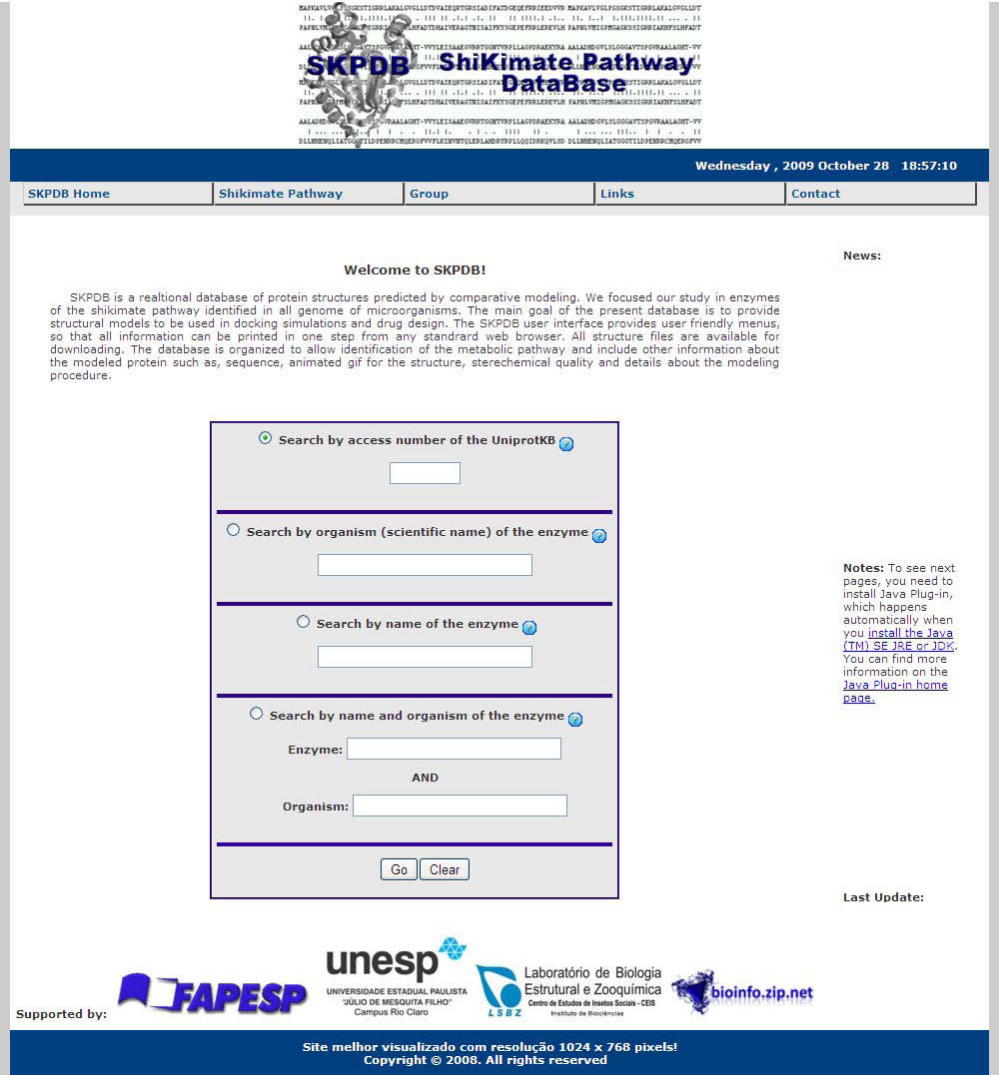
**Homepage of SKPDB**.

**Figure 6:**
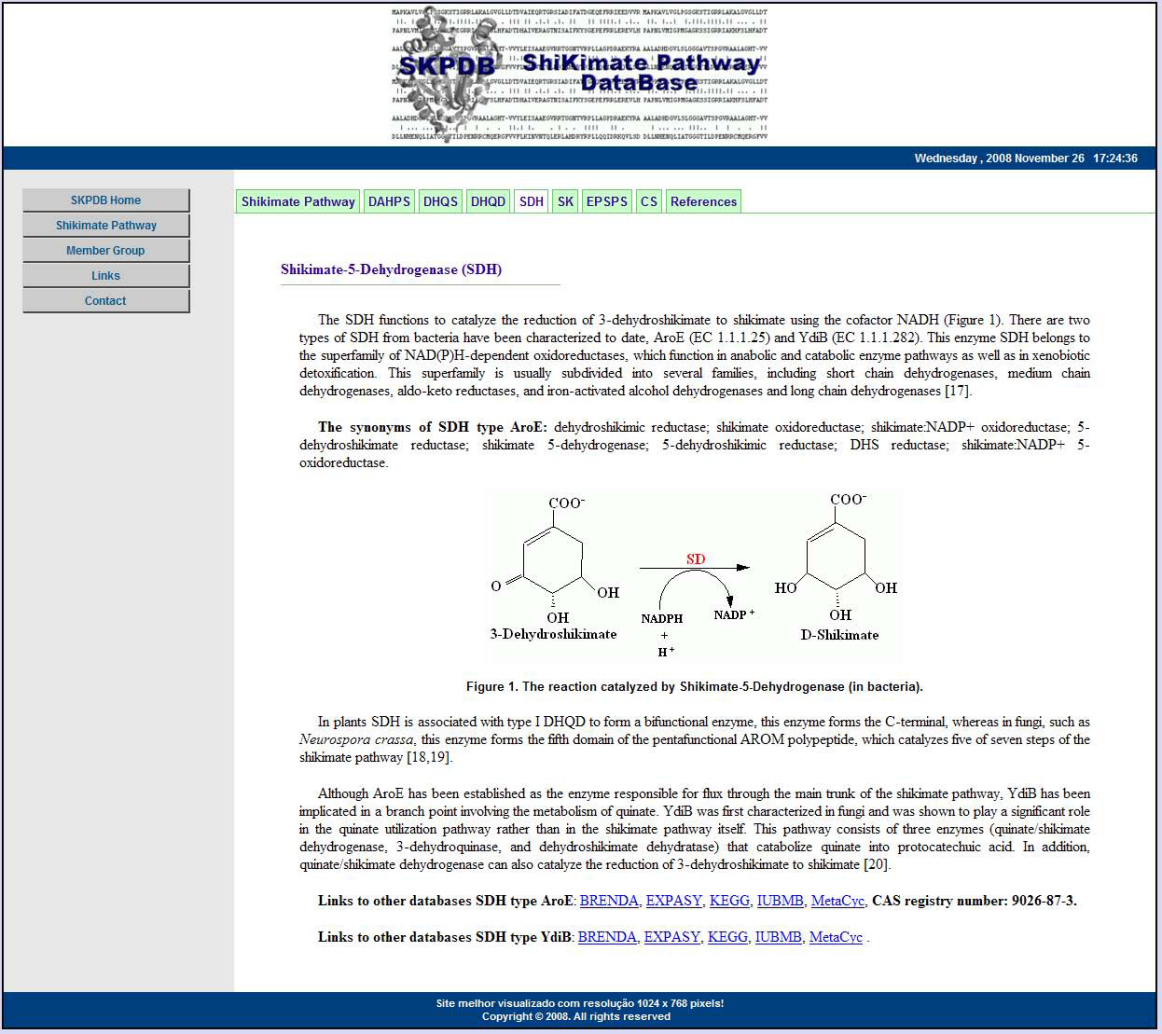
**The page for the shikimate pathway**.

The SKPDB provides a friendly interface with a menu and detailed help page for the searched function. In the menu on the left side of the homepage of SKPDB (Figure 5), users can find several information sources about the metabolic pathway of the shikimate pathway, with details about each enzyme (function, chemical structure, binding site, etc) displayed in figure 6. In addition, information about members of the group and contact details (an email for suggestions, criticisms and possible errors that the database might present) are provided.

### Searching tool in the SKPDB

The homepage offers to the user different ways for searching the database (Figure 5). The user can search in the SKPDB for a specific enzyme, just by using the Swiss-Prot+UniprotKB access number code. SKPDB also can be searched using the field "organism" or "name of enzyme", or even using a combination of both fields. A query is formed by selecting one radio button, and the blank text forms are entered with keywords or strings, such as a partial or full name of the enzyme or organism.

**Figure 7:**
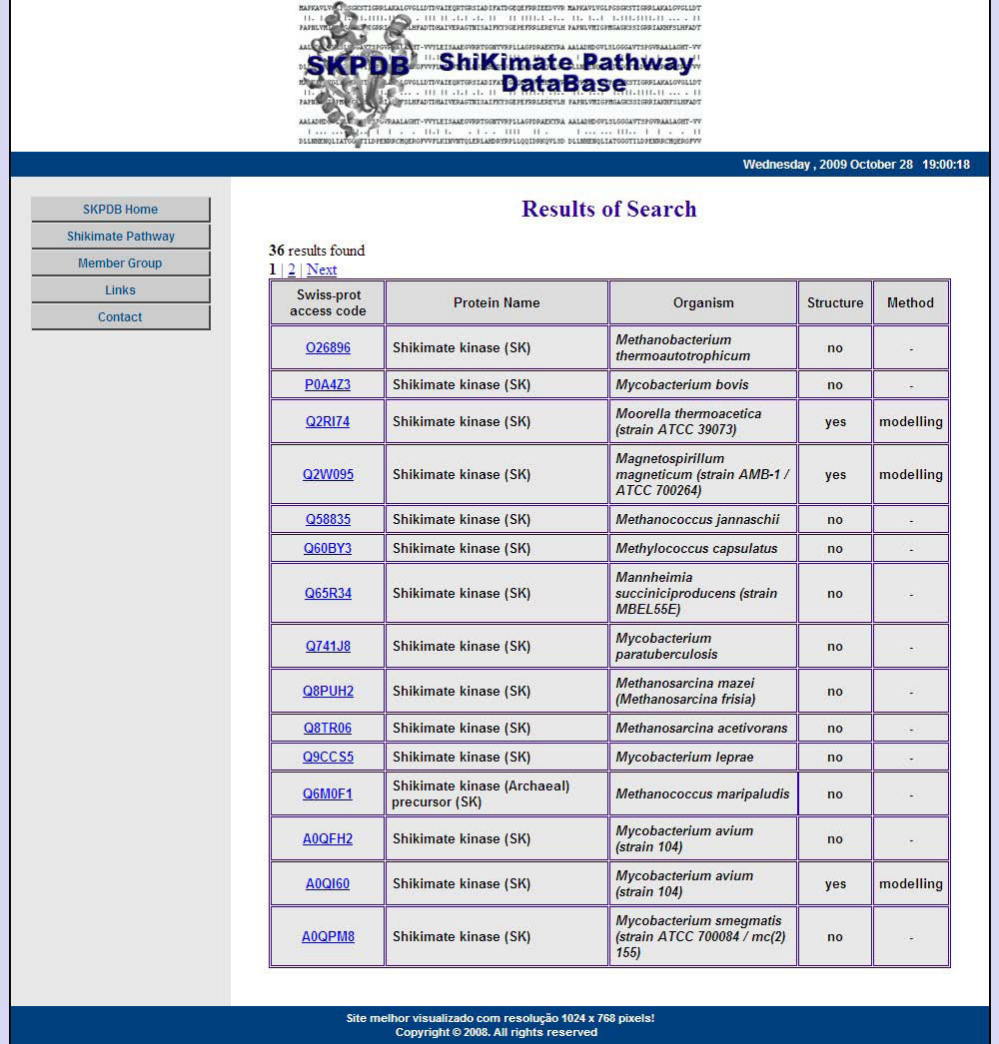
**This page is shown when the user searches by name of enzyme, or by organism, or by name of enzyme AND organism**.

**Figure 8:**
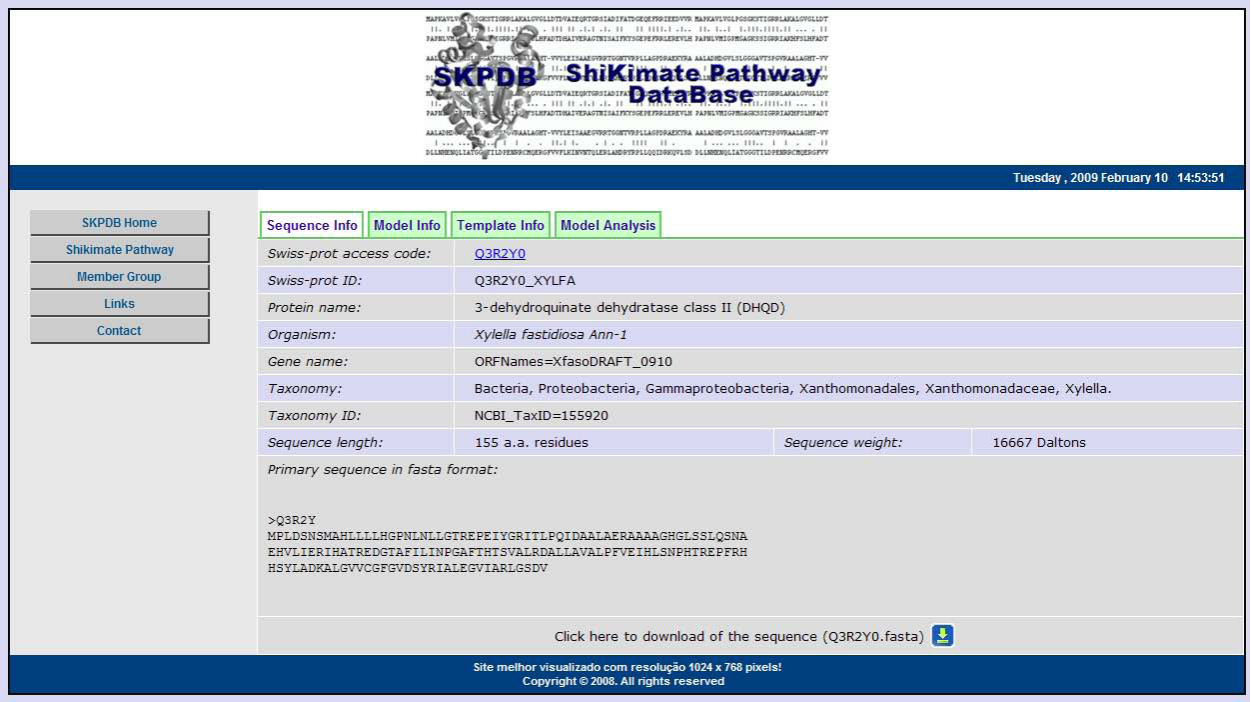
**The resulting page when the structure of the enzyme was predicted by molecular modeling**.

**Figure 9:**
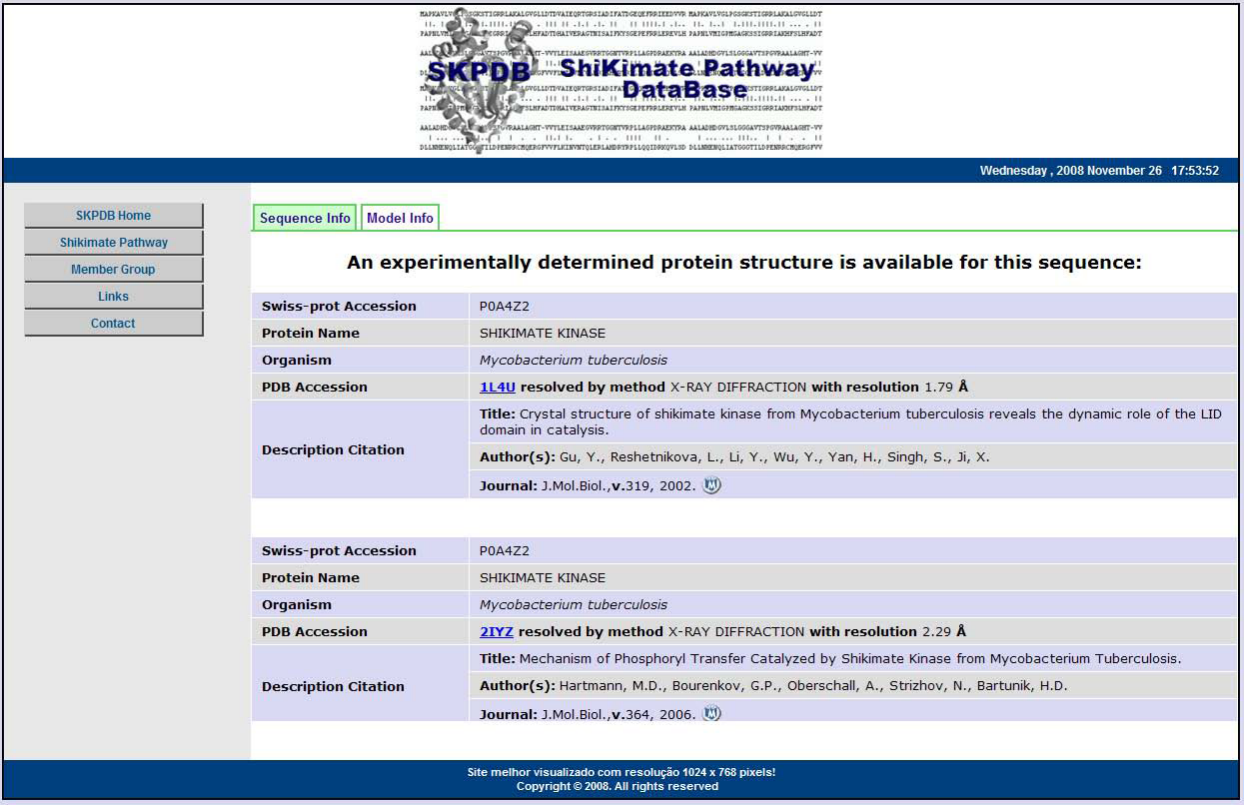
**The resulting page when the structure of the enzyme was experimentally determined**.

The HTML form parses the criteria into SQL database queries. The initial results returned are formatted into a summary table as described above (Figure 7). Selecting the access number of an enzyme in the summary table opens a new window displaying detailed information for the enzyme (Figure 8), if the structure was solved by molecular modeling. However, if the enzyme was resolved by NMR or crystallography, a page with information about the primary sequence and experimental data with external links to the PDB will be displayed (Figure 9).

When there is neither an experimentally determined structure nor templates to generate the homology model, a warning message is given.

### Output data

Output is generated in an html page too. The tab "Sequence Info" displays general information about the enzyme such as molecular weight, organism, taxonomy, size of the enzyme (aa), a download of the primary sequence, and a link to the Swiss-Prot+UniprotKB to increase accessibility to other types of information on the enzyme (Figure 8).

**Figure 10:**
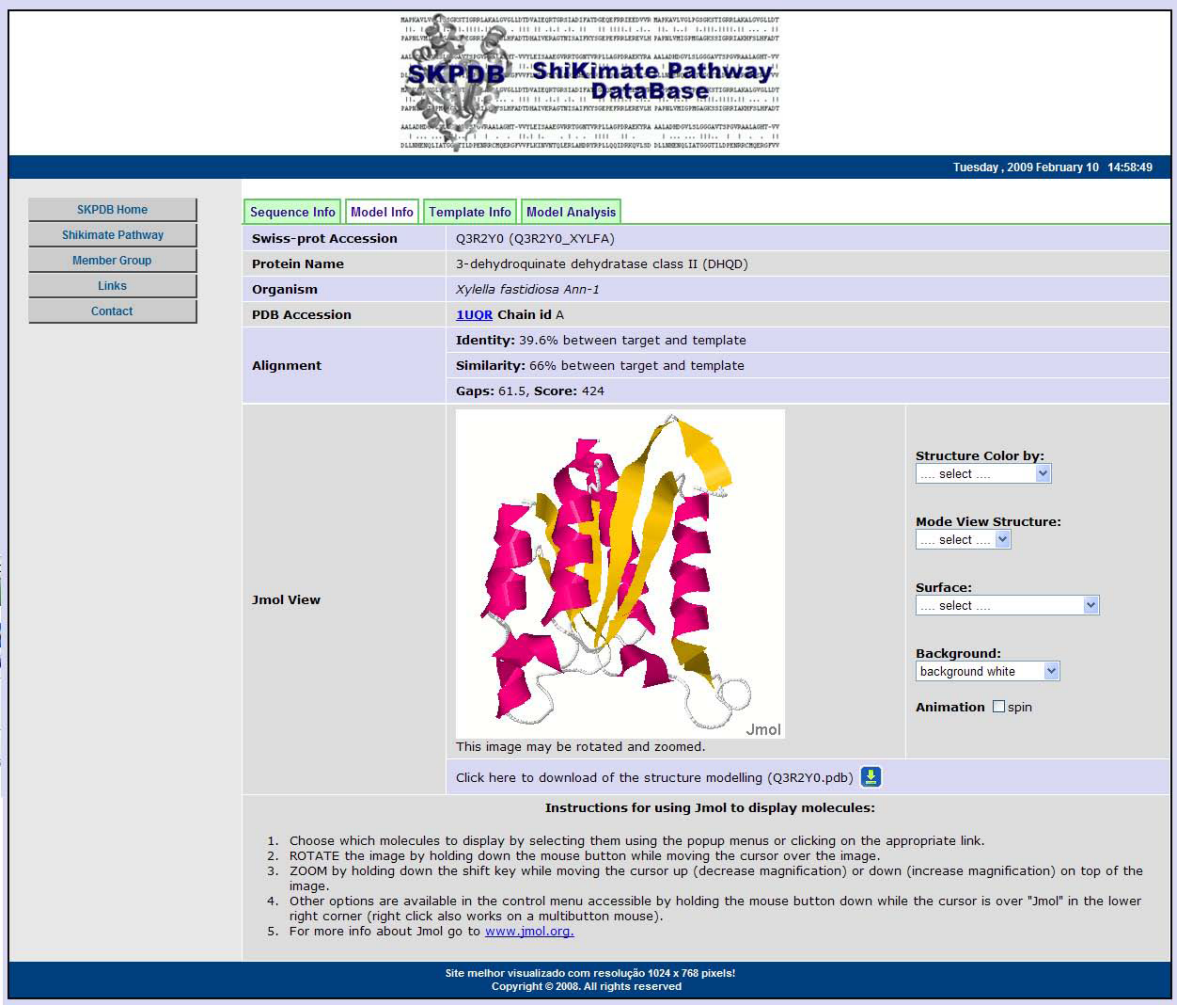
**The page "Model Info" has information about model predicted by molecular modeling**.

The tab "Model Info" has information about model predicted by molecular modeling, such as the template (PDB access code, the chain and an external link to the PDB) and the alignment (identity, similarity, and gaps score) between the primary sequences (target/template) and structure that can be viewed through Jmol. By default, structures in SKPDB are displayed as ribbons coloured by secondary structure. After loading, the user has several options that control the display of structure, selection of atoms and presentation styles. Users can also zoom in, zoom out, move and rotate the structure, or change the colour of the background. An example visualisation of the structure is shown in Figure 10.

**Figure 11:**
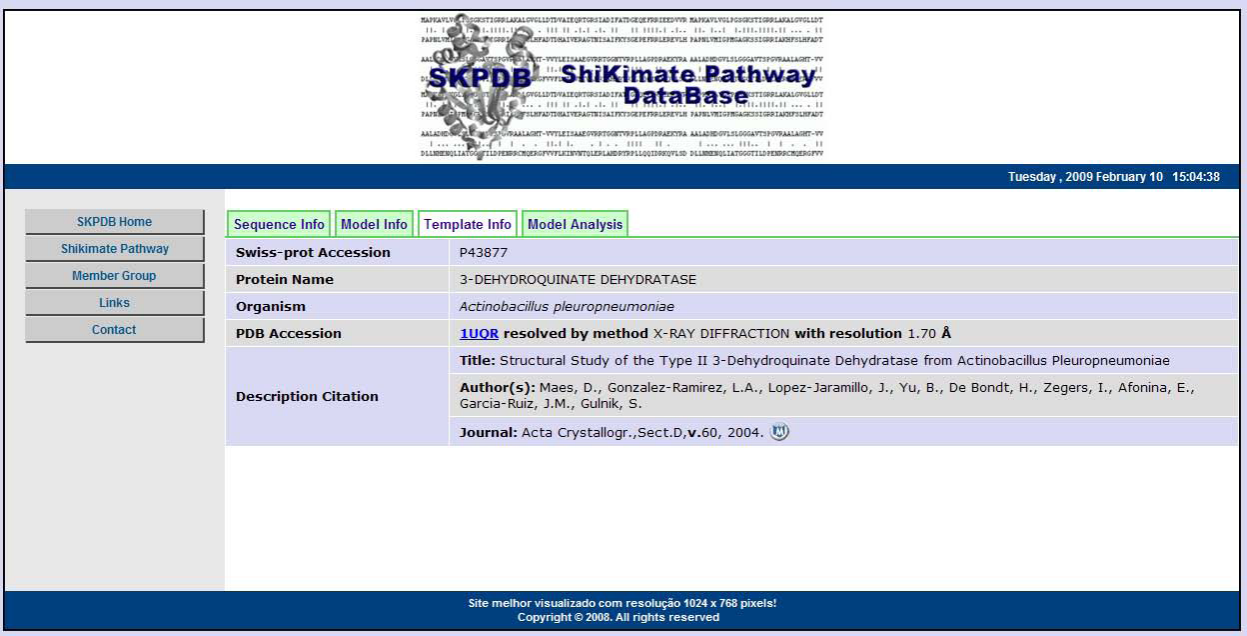
**The page "Template Info" has information about template used during molecular modeling**.

Information about template, organism, experimental method, resolution (if any) and, publication (if any - author, journal, title, volume, page, year and link to external access to PUBMED) are displayed in a tab called "Template Info", as shown in Figure 11.

**Figure 12:**
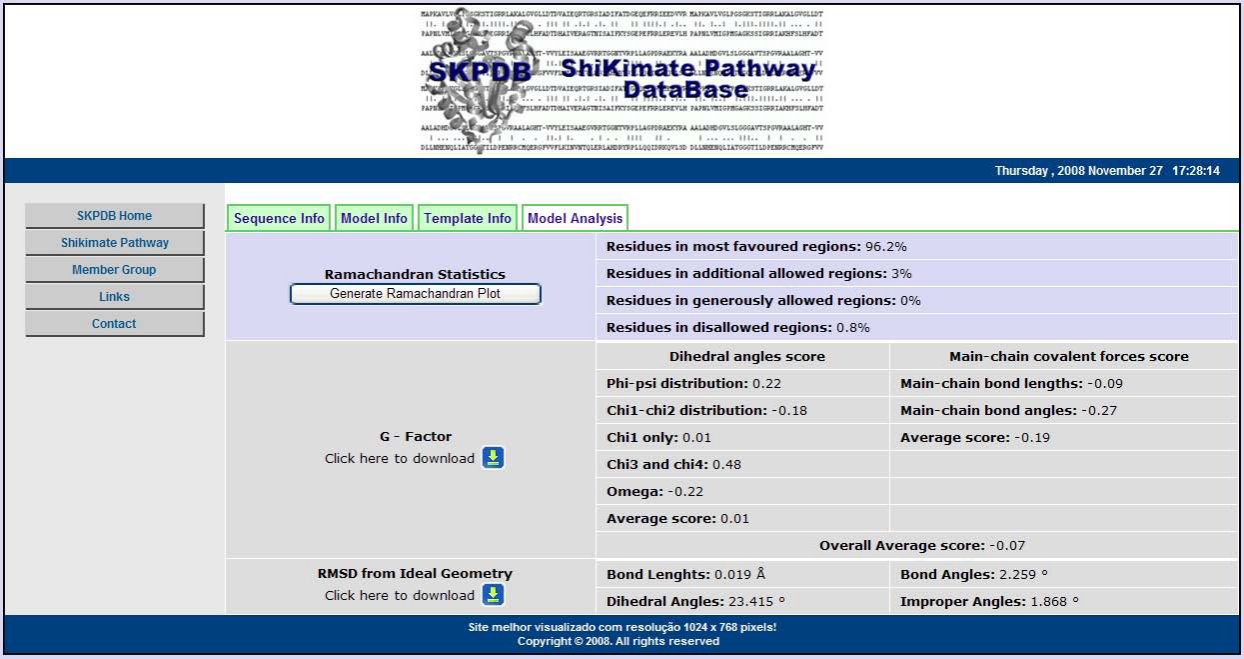
**The page "Model Analysis" has analysis of the models available dynamically in relation to the results of the generated model**.

Figure 12 shows the tab "Model Analysis" that presents the analysis of the models available dynamically in relation to the results of the generated model. This analysis provides web reports and charts of analysis of the chemical environment of the protein in addition to an analysis of the angles φ and ψ, establishes the position of the angles of twisting in the protein chain, checks possible stereochemical clashes between the side chains of atoms (PROCHECK and G Factor), and finally calculates the value of the RMSD from ideal geometry. All external links in SKPDB provide additional information with respect to the primary sequence and structural and functional annotations of the enzymes.

## Conclusions

Large scale protein homology modeling, in which whole sequence databases or whole genomes are used as inputs into automated modeling algorithms, has been reported by several groups [14,27]. By utilising powerful computer systems with multiple processors, these efforts have allowed the creation of large databases of homology models of proteins. This work resulted in the development of SKPDB, which is a useful tool in structural biology that provides annotating sequence information that contributes to structural biology and functional studies of shikimate pathway enzymes for drug development purposes. If a 3D model of the protein of interest can be derived, it may be usable as the basis for a structure-based drug-design study. In addition to this, such models can also be useful to the rational design of experiments such as site-directed mutagenesis.

## Availability and requirements

Project Home Page: http://lsbzix.rc.unesp.br/skpdb

Operating Systems: Linux Fedora

License: the SKPDB is publically accessible viacite

## Authors' contributions

HAA created the database and the automated annotation algorithm and wrote the manuscript. NJFS and CEB participated in the building of the database. GFDZ and EAM participated in building the database interface and suggestions in this manuscript. JMM conceived the study and suggestions. MSP and WFA conceived the study, and participated in its analysis and coordination. All authors read and approved the final manuscript.
